# Segregating Top-Down Selective Attention from Response Inhibition in a Spatial Cueing Go/NoGo Task: An ERP and Source Localization Study

**DOI:** 10.1038/s41598-017-08807-z

**Published:** 2017-08-29

**Authors:** Xiangfei Hong, Yao Wang, Junfeng Sun, Chunbo Li, Shanbao Tong

**Affiliations:** 1 Shanghai Key Laboratory of Psychotic Disorders, Shanghai Mental Health Center, Shanghai Jiao Tong University School of Medicine, Shanghai, 200030 China; 2 Shanghai Med-X Engineering Research Center, School of Biomedical Engineering, Shanghai Jiao Tong University, Shanghai, 200240 China; 3 School of Biomedical Engineering, Shanghai Jiao Tong University, Shanghai, 200240 China

## Abstract

Successfully inhibiting a prepotent response tendency requires the attentional detection of signals which cue response cancellation. Although neuroimaging studies have identified important roles of stimulus-driven processing in the attentional detection, the effects of top-down control were scarcely investigated. In this study, scalp EEG was recorded from thirty-two participants during a modified Go/NoGo task, in which a spatial-cueing approach was implemented to manipulate top-down selective attention. We observed classical event-related potential components, including N2 and P3, in the attended condition of response inhibition. While in the ignored condition of response inhibition, a smaller P3 was observed and N2 was absent. The correlation between P3 and CNV during the foreperiod suggested an inhibitory role of P3 in both conditions. Furthermore, source analysis suggested that P3 generation was mainly localized to the midcingulate cortex, and the attended condition showed increased activation relative to the ignored condition in several regions, including inferior frontal gyrus, middle frontal gyrus, precentral gyrus, insula and uncus, suggesting that these regions were involved in top-down attentional control rather than inhibitory processing. Taken together, by segregating electrophysiological correlates of top-down selective attention from those of response inhibition, our findings provide new insights in understanding the neural mechanisms of response inhibition.

## Introduction

As a hallmark of executive functions, response inhibition plays a crucial role in generating flexible and goal-directed behavior in everyday life^1^. It is often required in situations with immediate threats, for example, stopping crossing the street when the traffic signal turns red. In the laboratory, response inhibition paradigms, i.e., Go/NoGo and Stop-Signal tasks, have been widely used to investigate the underlying behavioral and neural mechanisms. In the standard version of the Go/NoGo task, participants are instructed to respond to a frequent “Go” stimulus while refraining from responding to an infrequent “NoGo” stimulus. The behavioral performance of response inhibition is usually represented by the error rates for NoGo stimuli. Neuroimaging techniques have been used to find differences in brain activity between NoGo and Go stimuli, to reveal the relevant neural substrates of response inhibition.

In spite of the many neuroimaging studies for response inhibition, there are still non-negligible controversies on its precise neural mechanisms. Successfully inhibiting a response tendency requires both inhibitory and non-inhibitory cognitive processes, such as attentional detection, conflict monitoring, working memory and error detection^2^. The influences of non-inhibitory cognitive processes on neural activity during response inhibition tasks could be elevated in experimental designs with relatively complex task requirements^3^. Therefore, the main challenge for studying response inhibition is to dissociate neural activities of the inhibitory process from those of non-inhibitory processes. Among those non-inhibitory processes, attentional detection, involving the identification of inhibition signals (e.g., NoGo or Stop signal) which cue response cancellation, is perhaps the most relevant because it is a prerequisite for successfully inhibiting a prepotent response tendency^4, 5^. In the Go/NoGo paradigm, NoGo stimuli are usually presented less frequently than Go stimuli in order to set up a prepotent response tendency and elicit a robust inhibitory process, which however, leads to the attentional capture of infrequent NoGo stimuli. Interestingly, recent neuroimaging studies have suggested that brain activation in the commonly reported candidate for response inhibition, i.e., the right inferior frontal cortices (RIFC), was largely driven by the attentional capture of infrequent stimulus, rather than inhibitory process per se^3, 6–13^.

It has been assumed that the infrequent stimuli for response cancellation captures participant’s attention in a stimulus-driven (bottom-up) manner^14^. However, according to the biased competition model of visual attention^15^, both top-down attentional modulations and bottom-up factors can affect stimulus processing. In experimental settings, Go and NoGo stimuli are usually presented in a fixed location where participant’s visual attention was already selectively deployed in a top-down manner, yielding a preparatory attentional state in anticipation of the forthcoming stimuli^16^. In this case, top-down attentional control plays a crucial role in the sensory detection of inhibition signals. Alternatively, one could imagine a situation with a stimulus outside the participant’s attended location, which may also serve as a signal for response inhibition. For example, a table tennis player will voluntarily cancel a prepared return either when the ball that flies within the player’s attended visual field is just out of bounds (need to discriminate between in and out before cancelling), or when the opponent errs and the ball flies outside the player’s attended visual field (no need to discriminate because the ball is definitely out). In the latter situation, top-down attentional modulation of the detection of inhibition signals should be kept at a minimal level, and the discrimination between Go and NoGo stimuli might not be necessary at all. Thus, our goal is to examine the differences in brain activity between these two types of response inhibition, and dissociate the neural activity of top-down attentional control from that of response inhibition.

**Figure 1:**
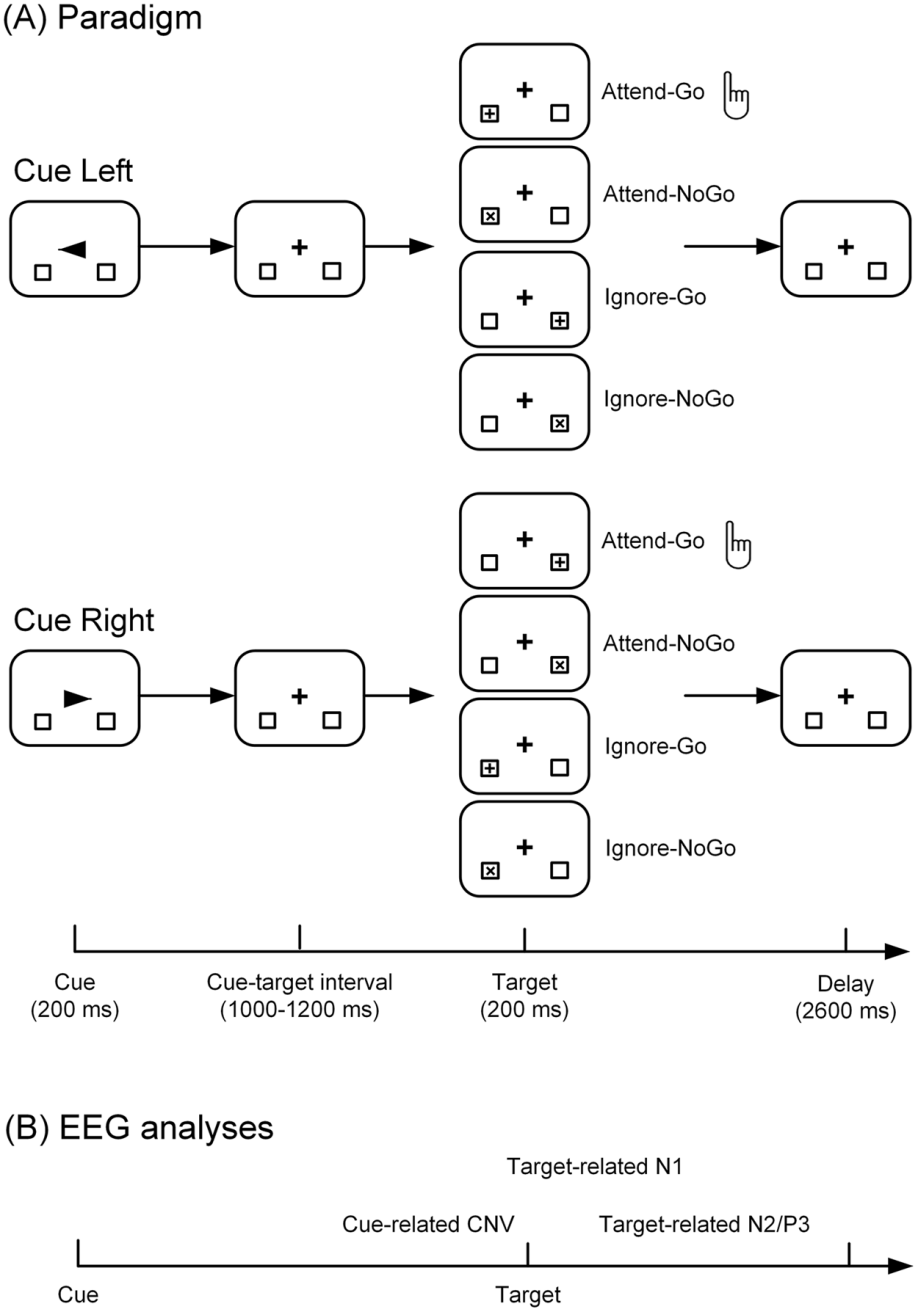
(**A**) Experimental paradigm. Participants were instructed to shift their visual attention covertly to either the left or the right location marker according to the direction of the arrow cue, and prepare for discrimination and response to the forthcoming targets. If the target was presented at the cued location, participants were required to discriminate between Go (the plus sign) and NoGo (the letter ‘x’), and respond via button-press to the Go target as quickly and accurately as possible. If the target was presented at the uncued location, participants were required to simply ignore it without any discrimination or motor response. (**B**) Time periods of ERP components that were analyzed in this study.

To accomplish this goal, we attempted to individually manipulate top-down selective attention and response inhibition in a modified Go/NoGo task combined with a spatial-cueing approach (Fig. 1A). The high temporal resolution of event-related potential (ERP) components derived from electroencephalography (EEG) enables us to examine the dynamic neural activity at the millisecond scale. Specifically, as illustrated in Fig. 1B, we expected to observe the contingent negative variation (CNV), N1, and N2/P3 components, which are associated with response preparation^17–19^, top-down attentional modulation^20, 21^, and response inhibition^22–24^, respectively. We hypothesized that if the neural activity of top-down selective attention and response inhibition could be segregated, there might be differences in the N2 and P3 components between the attended and ignored conditions of response inhibition (Fig. 1A). On one hand, response inhibition in the attended condition might recruit more neural resources in implicated brain regions (i.e., RIFC) and thus elicit larger P3 component than that in the ignored condition. On the other hand, the N2 component, which was recently argued to reflect conflict monitoring rather than inhibitory processing^24, 25^, might be suppressed or even eliminated in the ignored condition, as ignored targets are not actively discriminated and might not cause any conflict. Source localization was further performed to explore possible brain regions related to scalp-level ERP differences.

## Results

### Behavioral performance

The overall accuracy across all types of trials was 99.52 ± 0.06% (mean ± SEM, standard error of the mean), and the mean reaction time for Attend-Go targets was 469.66 ± 9.82 ms. Furthermore, the error rates were analyzed and compared between the attended (Attend-NoGo) and ignored (collapsed across Ignore-Go and Ignore-NoGo) conditions of response inhibition, and no significant difference was observed (attended condition: 0.19 ± 0.04% vs. ignored condition: 0.11 ± 0.03%; *t*_(29)_ = 1.82, *p* > 0.05). The negligible error rates (<1% on average) suggested that all participants correctly performed the task and showed successful response inhibition.

### ERP results

#### Cue-related CNV component

**Figure 2:**
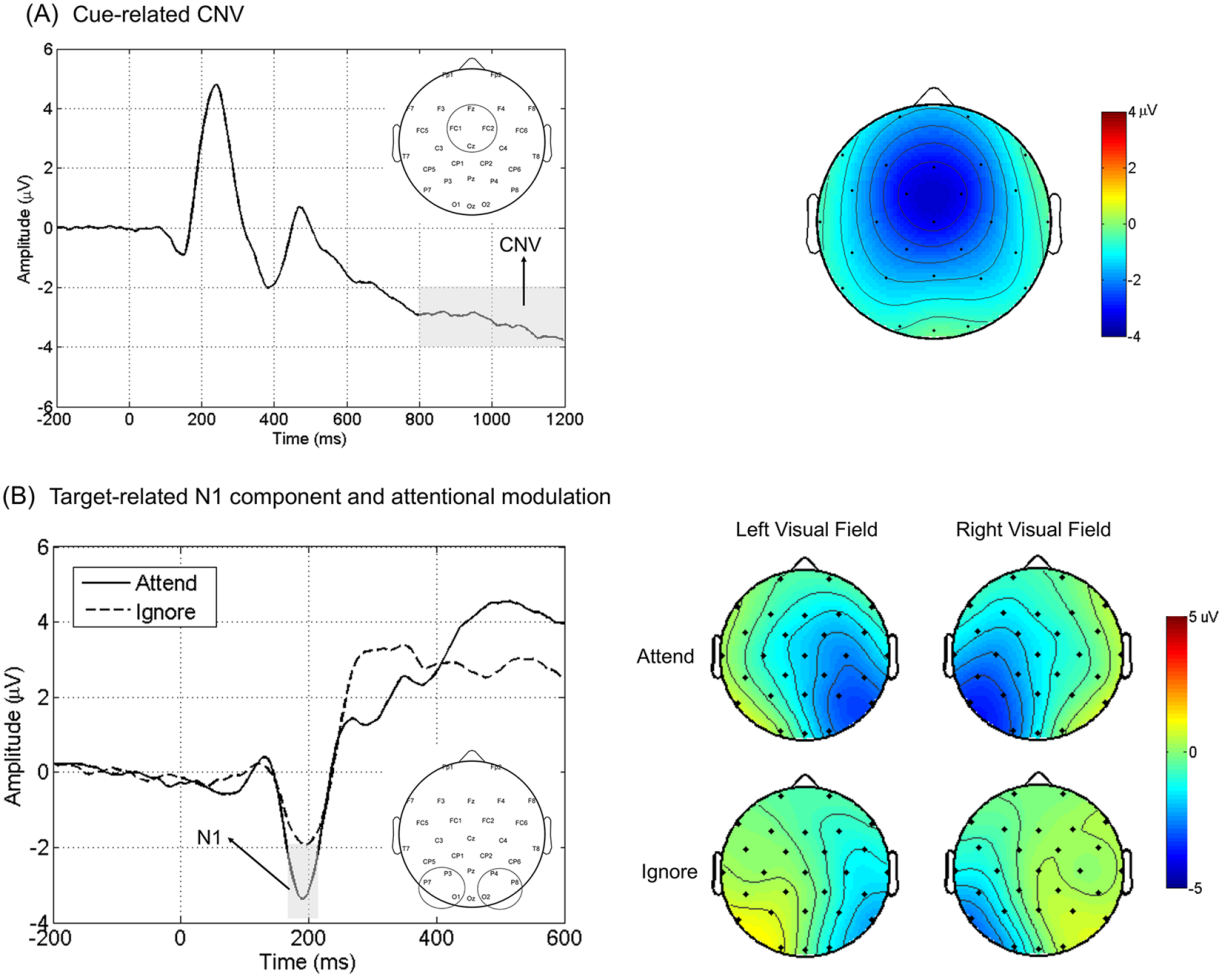
Cue-related CNV (**A**) and Target-related N1 (**B**). Panel A presents the grand-averaged ERP waveforms evoked by cues within the frontal-central ROI, collapsed for the left and the right cue trials. The topographical map of CNV averaged within 800–1200 ms post-cue interval is presented on the right side. Panel *B* illustrates the grand-averaged ERP waveforms evoked by attended versus ignored targets, which were averaged within the posterior ROIs contralateral to target location and collapsed across the left and right visual fields. The topographical maps of N1 component averaged within 170–210 ms post-target interval are presented on the right side.

Cue-related CNV (−3.19 ± 0.28 μV) was clearly observed in the ERP waveform averaged across the electrodes within the frontal-central region of interest (ROI, including Fz, Cz, FC1 and FC2; see Fig. 2A) during the late period of CTI (800–1200 ms post-cue), indicating the robust response preparation during the foreperiod.

#### Target-related N1 component

The early sensory ERP component (i.e., N1) enhanced by top-down selective attention is a robust electrophysiological marker for attentional modulation^20, 21, 26^. In this study, attentional modulation was indexed by the amplitude of target-related N1 component. To this end, all targets were grouped according to their location (the left or right visual field) and attention condition (attended or ignored). Then we averaged the target-related ERP waveforms within the contralateral posterior ROIs (Fig. 2B). The amplitudes of N1 were tested by a two-way ANOVA with Attention (attended, ignored) and Target Location (left visual field, right visual field) as within-subject factors. A main effect for Attention (*F*_(1,29) =  _62.786, *p* < 0.001) was observed, suggesting that attended targets elicited significantly larger N1 amplitudes than ignored targets. There were no other significant main effects or interactions observed.

#### Target-related N2 component

**Figure 3:**
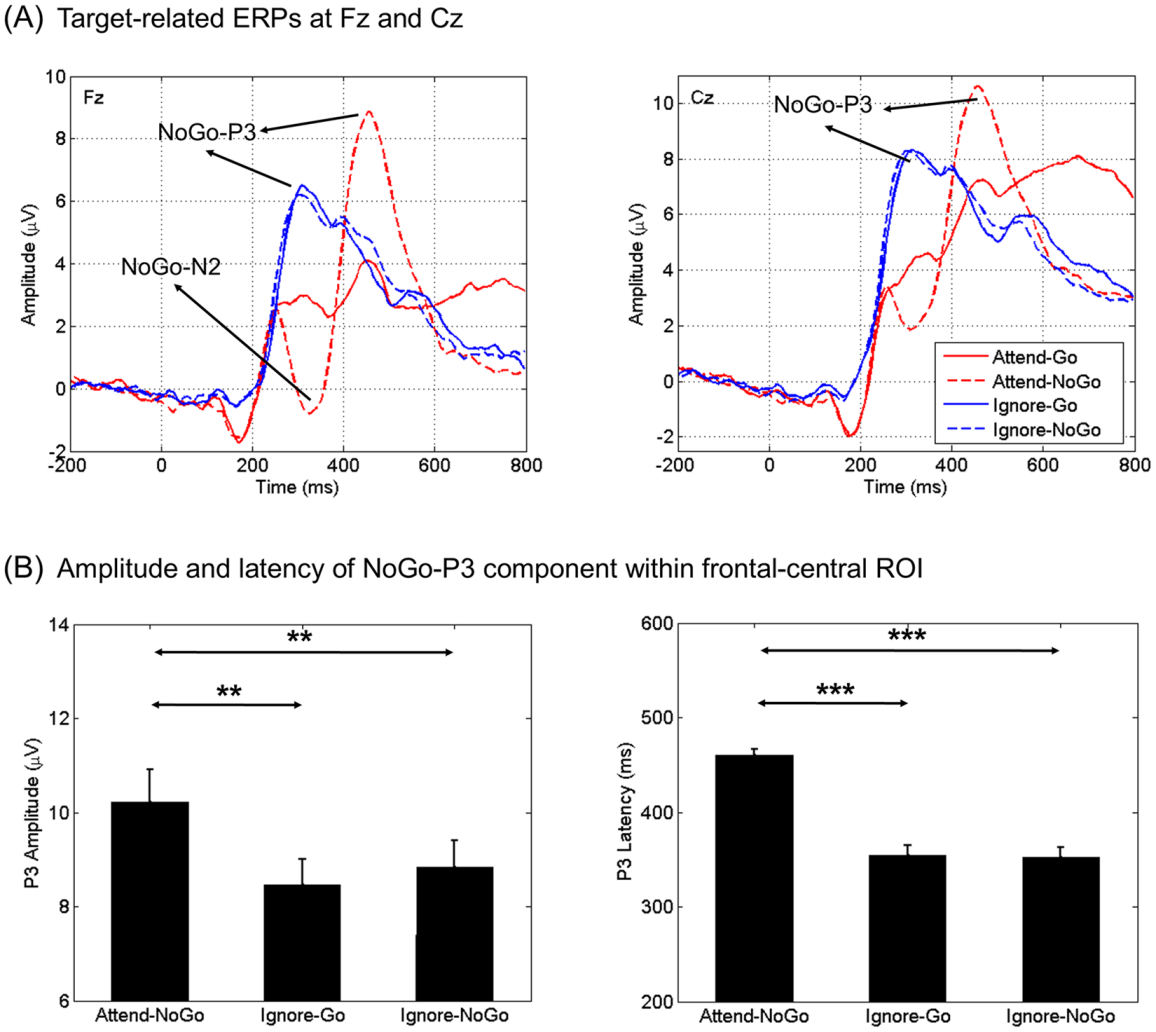
Target-related N2 and P3 components. Panel A illustrates the grand-averaged ERP waveforms evoked by different types of targets at Fz and Cz electrodes separately. Panel B shows the results of statistical comparisons of the amplitude and latency of P3 component between different types of targets (**p < 0.01; ***p < 0.001). Vertical bars indicate mean ± SEM.

**Figure 4:**
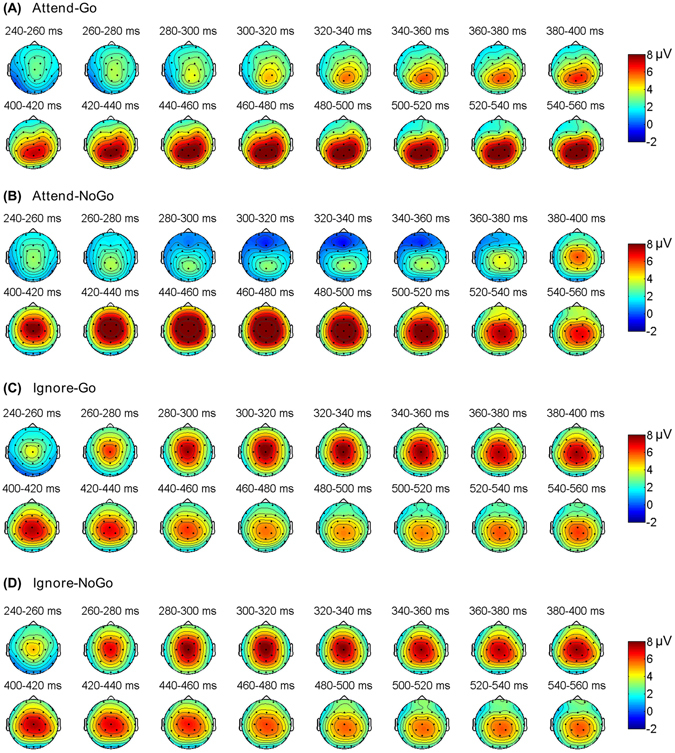
The grand-averaged topographical maps for N2 and P3 components, which were averaged within each successive 20 ms window between 240–560 ms post-target period for different types of targets.

As illustrated in Figs 3 and 4, a classical frontal N2 component was clearly visible for Attend-NoGo targets, while for ignored (Ignore-Go, Ignore-NoGo) targets, N2 was not observed. The N2 component evoked by Attend-NoGo targets was measured at the Fz electrode (amplitude: −1.71 ± 0.78 μV; latency: 319 ± 6 ms), and was found to be statistically significant (*t*_(29)_ = −5.235, *p* < 0.001) compared with the amplitudes of ERPs elicited by Attend-Go targets during the same time interval.

#### Target-related P3 component

For Attended-NoGo targets, we observed a classical frontal-central P3 component following the N2. For ignored targets, we found a similar P3 component with earlier peak latency, although the N2 was absent (Fig. 3). Moreover, the topography of P3 was similar between Attend-NoGo and ignored targets (Fig. 4). The P3 component was then measured in target-related ERP waveforms averaged across the frontal-central electrodes (Fz, Cz, FC1 and FC2), where the P3 has typically been observed in the literature^22–24^. The amplitudes of P3 were tested by a two-way ANOVA with Attention (attended, ignored) and Target (NoGo, Go) as within-subject factors. We observed a main effect for Target (*F*_(1,29) = _36.078, *p* < 0.001) and a two-way interaction for Attention × Target (*F*_(1,29) = _21.759, *p* < 0.001). Paired-samples *t*-tests were then performed to understand the interaction. Results showed that (Fig. 3B): (i) Attend-NoGo targets elicited larger P3 than Attend-Go (*t*_(29)_ = 5.605, *p* < 0.001), Ignore-Go (*t*_(29)_ = 3.761, *p* = 0.001) and Ignore-NoGo targets (*t*_(29)_ = 2.845, *p* = 0.008); (ii) There were no significant differences between Ignore-Go and Ignore-NoGo targets (*t*_(29)_ = −1.432, *p* = 0.163).

Additionally, we compared the latency of P3 between different types of targets. Results showed that (Fig. 3B): (i) Attend-NoGo targets elicited later P3 than Ignore-Go (*t*_(29)_ = 8.666, *p* < 0.001) and Ignore-NoGo targets (*t*_(29)_ = 9.093, *p* < 0.001); (ii) There were no significant differences between Ignore-Go and Ignore-NoGo targets (*t*_(29)_ = 0.147, *p* = 0.884). Since there were no significant differences in either amplitude or latency between Ignore-Go and Ignore-NoGo targets, these two types of targets were combined as Ignore targets in the following ERP analyses.

#### Correlation between CNV and N2/P3 components

**Figure 5:**
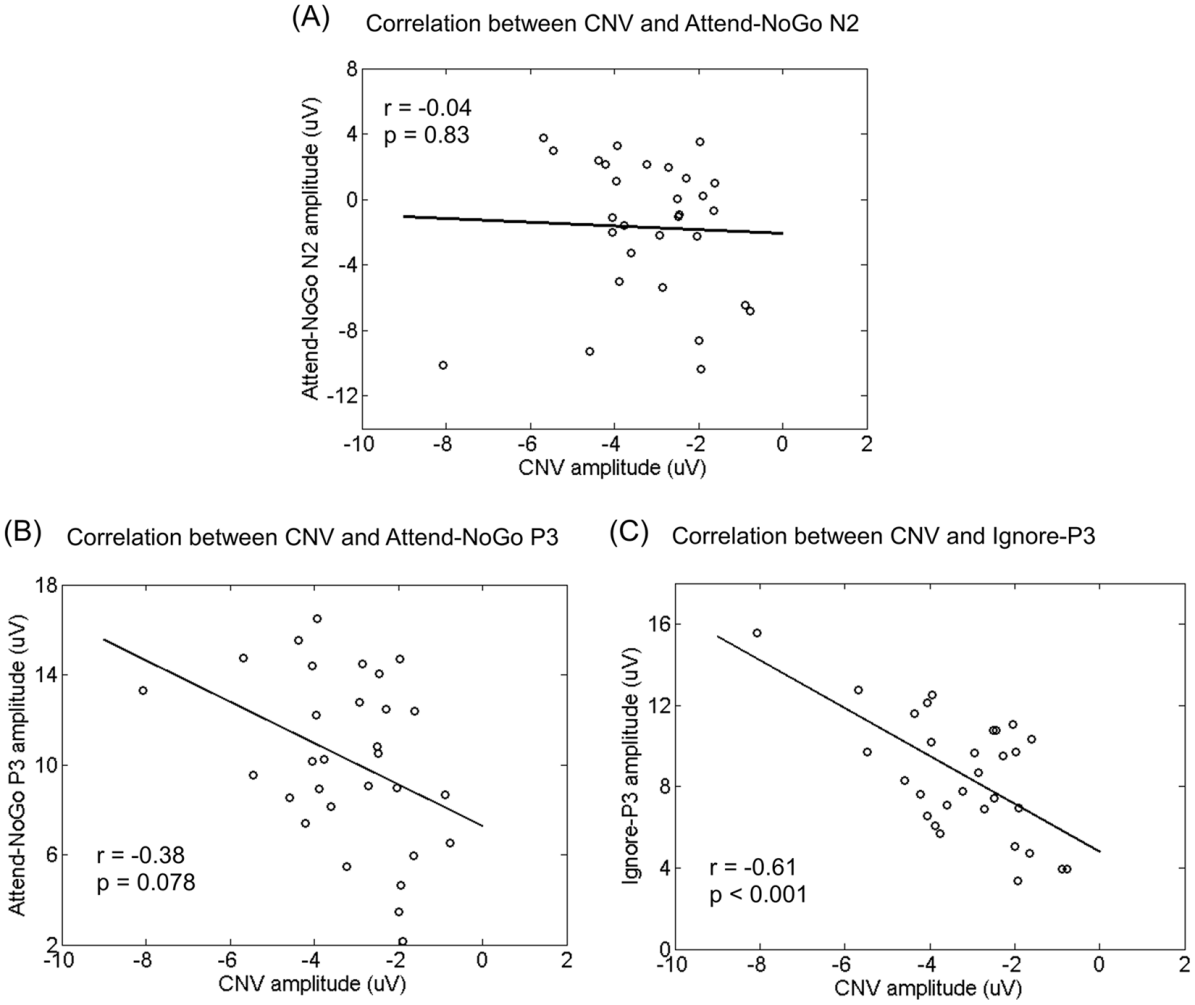
The Pearson correlation between CNV and N2/P3 amplitudes across all participants. All *p*-values were Bonferroni-corrected.

Since the CNV is a well-documented ERP marker for response preparation during the foreperiod^17–19, 27^, its correlation with the N2 or P3 component would help to reveal the functional significance of these two components. To this end, the amplitudes of CNV and N2/P3 components were analyzed by Pearson correlation on a subject-by-subject basis. The correlation was statistically significant between CNV and P3 for Ignore targets (Bonferroni-corrected *p* < 0.001, see Fig. 5C), and approached statistical significance for Attend-NoGo targets (Bonferroni-corrected *p* = 0.078, see Fig. 5B). For Attend-Go targets, there was no observed P3 and thus the correlation analysis was not performed. Furthermore, there was no significant correlation between CNV and N2 (for Attend-NoGo targets, see Fig. 5A; for other types of targets, there was no observed N2).

### sLORETA results

**Figure 6:**
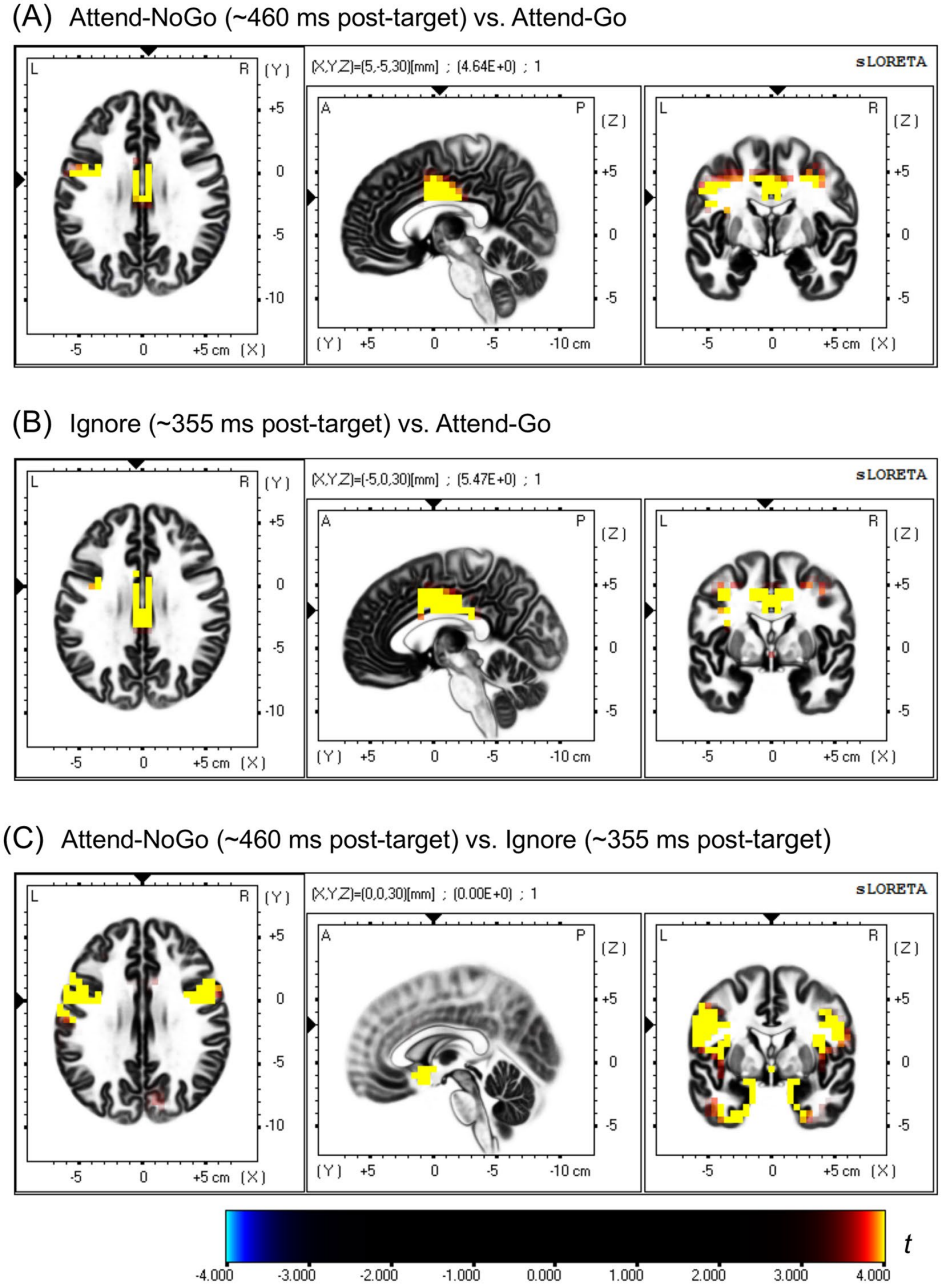
sLORETA-based Statistical non-Parametric Mapping (SnPM) of within-subject comparisons between different experimental conditions for the current density values of P3 component (Panel A: Attend-NoGo vs. Attend-Go; Panel B: Ignore vs. Attend-Go; Panel C: Attend-NoGo vs. Ignore). Each panel consists of slice views from axial, sagittal and coronal planes for the same brain area. The corresponding *t*-values of statistical significance (corrected *p* = 0.05, 2-tailed) are 3.909, 3.937 and 3.879 in A, B and C, respectively.

First, we analyzed the brain sources of the P3 component for Attend-NoGo targets by comparing source images between Attend-NoGo and Attend-Go targets. As illustrated in Fig. 6A, brain regions with significantly (corrected *p* < 0.05) greater activation for Attend-NoGo targets than Attend-Go targets were mainly located in the cingulate gyrus (midcingulate cortex), precentral gyrus, middle frontal gyrus and inferior frontal gyrus (Table 1). On the other hand, Attend-Go targets also evoked greater activation than Attend-NoGo targets in the superior parietal lobule, inferior parietal lobule and precuneus regions (Table 1).

**Table 1:** Brain areas that showed significant P3-related activations (2-tailed, corrected *p* < 0.05) for Attend-NoGo targets (~460 ms post-target) compared with Attend-Go targets. Only activation clusters including at least 5 voxels are shown in the table.

Brain structure	Brodmann area	Hemisphere	Lobe	Voxel number	Peak Coordinates MNI (X Y Z)			Peak t-value
***Attend-NoGo*** > ***Attend-Go***								
Cingulate Gyrus	23,24,32	L/R	Limbic	64	5	−5	30	4.6370
Precentral Gyrus	4,6	L/R	Frontal	40	35	−15	50	4.4556
Middle Frontal Gyrus	6	L/R	Frontal	12	35	−10	45	4.5606
Inferior Frontal Gyrus	6,9	L	Frontal	6	−45	0	35	4.3533
***Attend-NoGo*** < ***Attend-Go***								
Superior Parietal Lobule	7,40	L/R	Parietal	68	−30	−60	50	−5.1477
Inferior Parietal Lobule	7,39,40	L/R	Parietal	46	−35	−55	50	−4.8831
Precuneus	7,19	L/R	Parietal	26	−25	−60	50	−4.9365

Second, we compared the sources of the P3 component between Ignore-Go and Ignore-NoGo targets. No significant (corrected *p* < 0.05) differences were observed. Therefore, these two types of targets were combined as Ignore targets in the following source analyses, the same approach as used in ERP analysis. Then, we compared the brain sources of the P3 component between Ignore and Attend-Go targets. As illustrated in Fig. 6B, brain regions with significantly (corrected *p* < 0.05) greater activation for Ignore-targets than Attend-Go targets were mainly located in the cingulate gyrus (midcingulate cortex), precentral gyrus, middle frontal gyrus and postcentral gyrus (Table 2).

**Table 2:** Brain areas that showed significant P3-related activations (2-tailed, corrected *p* < 0.05) for Ignore targets (~355 ms post-target) compared with Attend-Go targets. Only activation clusters including at least 5 voxels are shown in the table.

Brain structure	Brodmann area	Hemisphere	Lobe	Voxel number	Peak Coordinates MNI (X Y Z)			Peak t-value
***Ignore*** > ***Attend-Go***								
Cingulate Gyrus	23,24,31,32	L/R	Limbic	114	−5	0	30	5.4713
Precentral Gyrus	4,6,9	L/R	Frontal	47	−35	5	40	4.8676
Middle Frontal Gyrus	6	L/R	Frontal	24	−35	0	40	4.8826
Postcentral Gyrus	3	R	Parietal	6	30	−25	45	4.2346
***Ignore*** < ***Attend-Go***								
No significant activation								

Third, we investigated the effects of top-down selective attention by comparing the sources between Attend-NoGo and Ignore targets. As illustrated in Fig. 6C, brain regions with significantly (corrected *p* < 0.05) greater activation for Attend-NoGo targets than Ignore targets were mainly located in inferior frontal gyrus, precentral gyrus, insula, uncus and middle frontal gyrus (Table 3).

**Table 3:** Brain areas that showed significant P3-related activations (2-tailed, corrected *p* < 0.05) for Attend-NoGo targets (~460 ms post-target) compared with Ignore targets (~355 ms post-target). Only activation clusters including at least 5 voxels are shown in the table.

Brain structure	Brodmann area	Hemisphere	Lobe	Voxel number	Peak Coordinates MNI (X Y Z)			Peak t-value
***Attend-NoGo > Ignore***								
Inferior Frontal Gyrus	6,9,13,44,45,47	L/R	Frontal	78	−50	0	25	5.5200
Precentral Gyrus	4,6,9,43,44	L/R	Frontal	69	−55	0	25	5.6391
Insula	13,47	L/R	Sub-lobar	61	35	0	20	4.8343
Uncus	20,28,34,36,38	L/R	Limbic	55	20	5	−25	4.8645
Middle Frontal Gyrus	6,8,9,46	L/R	Frontal	51	−50	5	40	4.6790
Parahippocampal Gyrus	28,34,35	L/R	Limbic	30	20	5	−20	4.9073
Subcallosal Gyrus	25,34,47	L/R	Frontal	19	15	5	−15	4.9924
Superior Temporal Gyrus	22,38	L/R	Temporal	17	25	10	−25	4.5455
Postcentral Gyrus	1,3,40,43	L	Parietal	13	−55	−10	20	4.9435
Rectal Gyrus	11	L/R	Frontal	13	10	15	−25	4.7966
Anterior Cingulate	25,32	L/R	Limbic	13	5	10	−10	4.7686
Medial Frontal Gyrus	11,25	L/R	Frontal	12	15	10	−20	4.9861
Inferior Temporal Gyrus	20	L	Limbic	12	−30	−5	−45	4.3646
***Attend-NoGo < Ignore***								
No significant activation								

**Figure 7:**
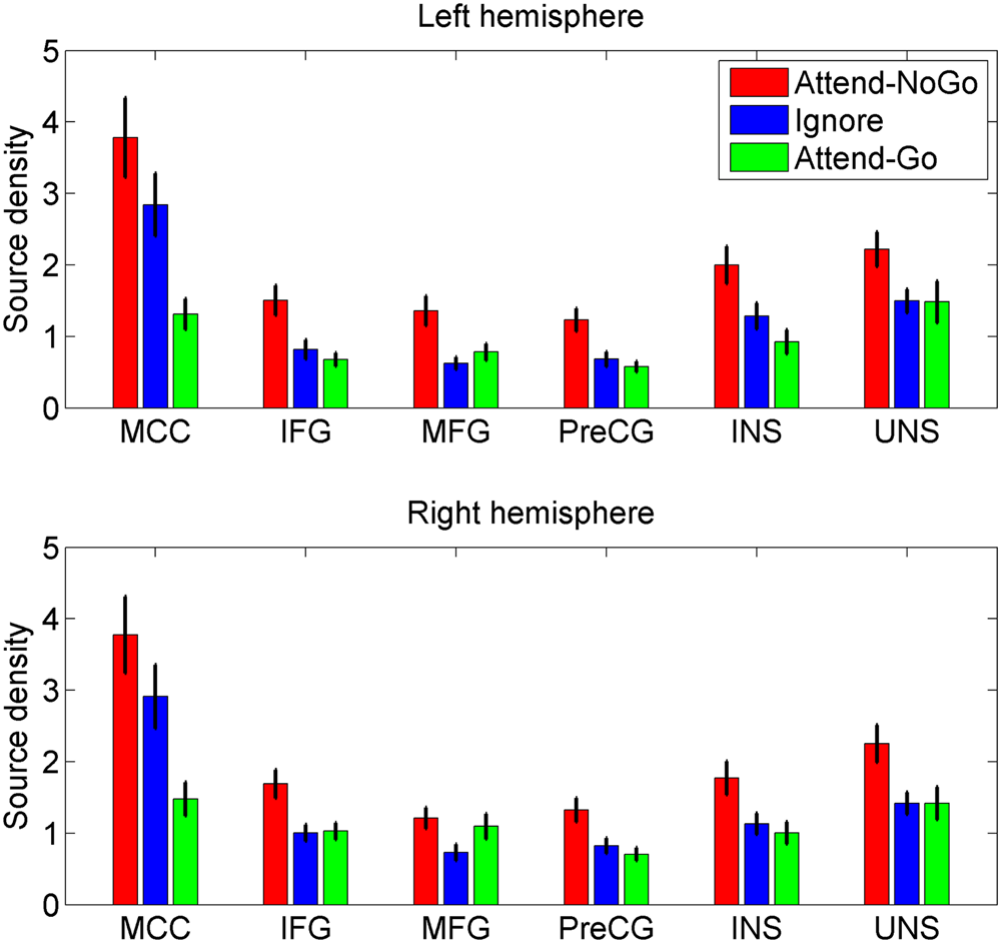
The grand-averaged source density in the midcingulate cortex (MCC), inferior frontal gyrus (IFG), middle frontal gyrus (MFG), precentral gyrus (PreCG), insula (INS) and uncus (UNS) for different experimental conditions. Vertical bars indicate mean ± SEM.

Furthermore, we identified several brain regions based on the above results, including the midcingulate cortex (MCC), inferior frontal gyrus (IFG), precentral gyrus (PreCG), insula (INS), uncus (UNS) and middle frontal gyrus (MFG), and reconstructed their source densities using the sLORETA software, for the left and right hemispheres separately. As illustrated in Fig. 7, only in the MCC, both Attend-NoGo and Ignore targets evoked clearly greater activation than Attend-Go targets. In the rest regions, however, only Attend-NoGo, but not Ignore targets, showed obviously greater activation than Attend-Go targets.

In summary, our results showed that the MCC was the primary source for the P3 component. Moreover, compared with Ignore targets, Attend-NoGo targets elicited greater activation in several frontal and limbic regions, including IFG, MFG, PreCG, INS, and UNS.

## Discussion

In this study, we successfully manipulated top-down selective attention and response inhibition using a spatially cued Go/NoGo task, as indicated by the classical ERP components, i.e., CNV, N1, N2 and P3, observed in our results. Since our main objective was to segregate the neural correlates of top-down selective attention from those of response inhibition, we compared the ERP activity between Attend-NoGo targets (which required both attention and inhibition) and Ignore targets (which required inhibition, but not attention). Our key findings were differences in both scalp-level ERPs and source-level activities between Attend-NoGo and Ignore targets. Our results highlight the importance of segregating the neural activity of top-down selective attention when investigating the neural mechanisms of response inhibition.

The cued Go/NoGo task has been adopted to investigate the neural mechanisms of response inhibition in previous studies, in which cues were used to manipulate the levels of response preparation^18, 19, 28^. Similarly, a spatial-cueing approach was used in a Go/NoGo paradigm in this study (Fig. 1). Such a design could not only elicit the robust response preparation (as indicated by the CNV, see Fig. 2A), but also manipulate top-down selective attention (as indicated by the N1, see Fig. 2B). Moreover, the simple task setting in the present study (as indicated by the high accuracy rates and negligible error rates) was helpful to minimize the possible interferences from other complicated cognitive processes, such as working memory, which could be more confounding under complex settings^3^. One concern here is that the inhibition requirements might be reduced in such an easy task, which however, is not supported by our results. Specifically, the prominent CNV in reaction to the instructional cues clearly suggested a robust process of response preparation during the foreperiod (Fig. 2A), and the following N2 and P3 components clearly suggested a robust process of response inhibition (discussed later). Taken together, our ERP results, including the CNV, N1, N2 and P3, suggested that the manipulation of top-down selective attention and response inhibition was successful.

Although early studies usually regarded both the N2 and P3 components as the neural correlates of response inhibition^29–31^, recent research suggested that only the P3 may reflect inhibitory processing, while the N2 is more likely to reflect conflict monitoring between competing responses^18, 24, 25, 32^. Our findings provided further evidence for such an interpretation from two aspects. First, we found that only Attend-NoGo, but not Ignore targets, evoked the N2. Such a finding confirmed our hypothesis that Ignore targets were not attended or discriminated and thus caused no conflicts, supporting the role of the N2 as conflict monitoring rather than inhibitory processing. In contrast, the P3 component observed following both Attend-NoGo and Ignore targets clearly supported its inhibitory role, as participants should prepare for response regardless of the types of the forthcoming targets. Second, the CNV, a measure of response preparation during the foreperiod, demonstrated a significant correlation with the P3 (but not the N2), further supporting an inhibitory role for P3, but not N2^17–19, 27^.

The main point of interest of this study focused on the differences in the P3 between Attend-NoGo and Ignore targets. On one hand, we found that Ignore targets evoked a P3 component with similar topographical distribution but smaller amplitude than that evoked by Attend-NoGo targets. As participants maintained a central fixation during the task and trials where fixation was lost were rejected, differences in P3 amplitudes can be attributed primarily to differences in covert attention between targets. This finding confirmed our hypothesis that the ignored condition of response inhibition might recruit less neural resources than the attended condition due to a reduced requirement of top-down attentional control. More interestingly, it seemed that the CNV was more correlated with the P3 for Ignore targets (Fig. 6C, *r* = −0.61, Bonferroni-corrected *p* < 0.001) than that for Attend-NoGo targets (Fig. 6B, *r* = −0.38, Bonferroni-corrected *p* = 0.078), implying that the inhibitory processing following Ignore targets was less influenced by non-inhibitory cognitive processing, i.e., top-down selective attention. On the other hand, the latency of the P3 evoked by Attend-NoGo targets (~460 ms post-target) preceded the N2 (~319 ms post-target) around 141 ms, which was highly consistent with the literature^23, 24^. For Ignore targets, however, the latency of the P3 (~355 ms post-target) was significantly shorter due to the absence of the N2, suggesting that inhibitory processing was activated around 100 ms earlier (355 ms vs. 460 ms post-target) for Ignore targets than that for Attend-NoGo targets.

Although the source of the P3 in both Attend-NoGo and Ignore conditions was mainly located in the MCC (discussed later), the differences between these two conditions were located in several other brain regions, which mainly included the IFG, MFG, PreCG, INS and UNS (see Table 3 and Fig. 6C). Furthermore, by reconstructing source density in these regions (Fig. 7), we found that only MCC showed inhibition-related activation in both conditions, while the rest regions were sensitive to top-down selective attention rather than response inhibition. Specifically, for these regions, higher levels of activation were observed for the Attend-NoGo than the Attend-Go condition, while similar levels of activation were observed between the Ignore condition and the Attend-Go condition. Interestingly, these regions with greater activation in the Attend-NoGo condition were consistent with a previous fMRI study, in which similar brain regions were activated by attended visual targets in a top-down spatial-cueing attention task^33^. Thus, the present finding implied that the activation of these brain regions might be more related to top-down attentional control rather than inhibitory processing.

These implicated brain regions, especially the IFG, MFG and INS, have been widely credited with roles supporting response inhibition by numerous neuroimaging^34–36^ as well as lesion studies^37, 38^. However, a few studies challenged this view by showing that brain activation during response inhibition could be largely dependent on non-inhibitory cognitive demands, i.e., stimulus-driven attentional capture of infrequent stimuli^7, 8, 10, 11^, and it was proposed that there were no unique modules within the frontal lobes dedicated to response inhibition^9, 12^. Furthermore, a recently proposed theory that attempts to unify the literature on response inhibition, performance monitoring, attention, and working memory, argues that unexpected events could recruit a fronto-basal-ganglia network for motor inhibition. A key hypothesis of this theory is that motor inhibition and attentional orienting are independent processes^39^. This idea has been supported by a study that brain components for inhibitory control and stimulus-driven attentional orienting could be dissociated by comparing the EEG activity between a simple and a complex version of the stop-signal task^40^. Consequently, our findings that the neural activities for top-down attentional orienting could also be segregated from those of inhibitory processing, provide new implications for such a unifying theory of response inhibition^39^.

The MCC has frequently been reported to play a primary role in the generation of P3 component, while other areas, i.e., precentral cortex, middle frontal cortex, inferior frontal cortex and insula, have often been reported as contributing regions^23, 41–43^. Notably, recent studies that utilized EEG-informed functional magnetic resonance imaging (fMRI)^44, 45^ or MRI-guided EEG source modelling^43^ emphasized the important role of MCC in the generation of the P3. In this study, the dominant sources of the P3 for both Attend-NoGo (Table 1) and Ignore targets (Table 2) were located in the MCC, which was consistent with the literature. Furthermore, the comparison between Attend-NoGo and Ignore targets did not reveal any significant differences in the MCC (Table 3), which also implied that the MCC was activated by inhibitory processing rather than top-down selective attention.

In addition, it was worth mentioning the brain activation that was stronger for Attend-Go targets than Attend-NoGo targets (Table 1). This finding was not surprising, because Attend-Go targets elicited a central-parietal Go-P3 component (Fig. 4A). This P3 component usually reflects resources allocation or context updating, and might be generated in the parietal cortex^46–49^. In this study, although the comparison of source activities between Attend-NoGo and Attend-Go targets was conducted around the latency of the NoGo-P3, the Go-P3 should also be covered, at least to some extent, due to the obvious overlapping of these two types of P3 components. Nonetheless, since this study focused on the inhibition-related NoGo-P3 component, we did not examine brain regions related to the Go-P3 component.

To conclude, by manipulating top-down selective attention and response inhibition in a spatial cueing Go/NoGo task, we segregated the electrophysiological correlates of top-down selective attention from those of response inhibition. Our results clearly suggest that the influences from top-down selective attention should be taken into account when studying the neural substrates of response inhibition.

## Materials and Methods

### Participants

32 students (age 18–25 years, 12 females, all right-handed) from Shanghai Jiao Tong University were recruited in this study. All participants reported normal or corrected-to-normal vision, and no history of neurological or psychiatric disorders. Two participants were excluded from data analysis due to poor data quality (more than 50% trial rejection rate after preprocessing). The final sample included 30 participants (age 18–25 years, mean 21.33 years ± 1.94 s.d.; 12 females, all right-handed). Informed consent was obtained from each participant before the experiment. All participants were financially compensated after the experiment. The experiment protocols were approved by the institutional ethical committee of Shanghai Jiao Tong University, and complied with the Declaration of Helsinki.

### Stimuli and procedures

We combined a spatial-cueing approach with the Go/NoGo task to manipulate the top-down selective attention and response inhibition (Fig. 1A). Specifically, participants were required to deploy visual attention to the cued location covertly and prepare for motor response to the forthcoming targets. On one hand, when the target appeared at the attended location, participants were required to discriminate between Go and NoGo targets and execute the prepared response (Go) or inhibit it (NoGo). On the other hand, when the target appeared at the ignored location, participants were simply required to ignore it and inhibit the prepared response, regardless of its type (Go or NoGo). Such an experimental paradigm has been successfully adopted to study top-down selective attention and response inhibition by us refs 20, 22, 50 and others^26^. However, none of these studies explicitly attempted to segregate the neural activity of top-down selective attention from response inhibition.

Participants were comfortably seated in a sound attenuated room during the experiment. All stimuli were presented on a 19-inch LCD display (Dell: P190SB) placed 60 cm in front of the participant. During the experiment, a black central crosshair (1.38° by 1.38°) and two lateral black location markers (2.39° by 2.39°) were always presented with a white background. Participants were required to always maintain a central fixation throughout the experiment. In each trial, a black arrow cue was first presented centrally for 200 ms to instruct the subject to fully deploy covert attention to the cued location, and totally ignore other locations. The arrow cue pointed to the left or right location marker randomly (each with 50% probability) across trials. A black target (1.67° by 1.67°), either the plus sign or letter ‘x’, was presented for 200 ms at either the left or right location marker following a cue-target interval (CTI: 1000–1200 ms from cue offset to target onset). Participants were required to respond to the plus sign that was presented at the attended location (Attend-Go targets) with the right index finger as quickly and accurately as possible, and refrain from responding to the letter ‘x’ presented at the attended location (Attend-NoGo targets). Participants were also instructed to refrain from responding to both the plus sign (Ignore-Go targets) and letter ‘x’ (Ignore-NoGo targets) when presented at the ignored location (Fig. 1A). Both location and type of the targets were varied randomly with 50% probability. A fixed inter-trial interval of 2600 ms was presented between the target offset and the cue onset of next trial. For Attend-Go targets, only responses made within 1600 ms after the target offset were recorded as valid trials.

The experimental paradigm was implemented in E-Prime (Version: 2.0), and behavioral responses were recorded with the response box included in the E-Prime toolkit. Each block contained 60 trials and lasted for about 5 minutes. Each participant was trained for at least one block to get familiar with the experimental instructions, then completed 8 formal blocks with a 2–3 min break between two successive blocks, resulting in 480 trials for each participant.

### EEG recording

Continuous EEG signals were recorded during the experiment using a BrainAmp MR Plus amplifier and a 32 channel (recording channels: Fp1, Fp2, F3, F4, F7, F8, Fz, FC1, FC2, FC5, FC6, C3, C4, Cz, T7, T8, CP1, CP2, CP5, CP6, P3, P4, P7, P8, 171 Pz, O1, O2, Oz, TP9, TP10; recording reference: FCz; ground: AFz) EasyCap^TM^ (Brain Products GmbH, Gilching, Germany). Two additional electrodes were placed on the outer left canthus and above the right eye to record horizontal and vertical electrooculograms (EOGs), respectively. The signals were amplified and digitized at 1000 Hz sampling rate with 0.016–100 Hz online band-pass filtering. Impedance of each electrode was maintained below 10 kΩ during the recording.

### EEG preprocessing

EEG preprocessing was performed in Matlab-based (Version: R2014a) EEGLAB^51^ (Version: 13.5.4b) and ERPLAB^52^ (Version: 6.0) toolboxes. Raw EEG data first went through a band-pass filter between 0.1–40 Hz (a two-way Butterworth filter with zero phase shift; roll-off slope: 12 dB/oct) and a Parks-McClellan notch filter at 50 Hz. Ocular artifacts were corrected by independent component analysis based on the Infomax algorithm^53^. After that, continuous EEG data were down-sampled to 250 Hz and re-referenced to the algebraic average of the two mastoid electrodes (TP9 and TP10). Continuous EEG data were then segmented into two types of epochs: cue-related epochs (−200–1200 ms post-cue) and target-related epochs (−200–800 ms post-target). Artifact detection using ERPLAB were performed for all EEG epochs, including: (i) the examination of maximally allowed amplitude difference (threshold: 150 μV) for all EEG channels within a moving window (width: 200 ms; step: 50 ms) using the peak-to-peak function; (ii) the examination of maximally allowed absolute amplitude (threshold: ± 100 μV) for all EEG channels throughout the whole epoch. Furthermore, to ensure that participants maintained a central fixation and did not miss the stimuli, two additional steps were performed based on EOGs to identify EEG epochs with overt eye movements during the entire epoch or blinks during stimulus presentation: (i) the detection of eye movements in the HEOG channel using a step function with a moving window (width: 400 ms; step: 10 ms; threshold: 40 μV); (ii) the detection of eye blinks in the VEOG channel using a step function around the cue or target stimuli presentation period (−200–200 ms) with threshold of 50 μV. All EEG epochs were then visually inspected to ensure the quality before subsequent analysis. Two (out of 32) participants were excluded in the following analysis because of more than 50% rejection rate for cue-related epochs. After artifact rejection, the trial acceptance rate for the final sample (*N* = 30) was 86.16% ± 13.47% (mean ± s.d.) for cue-related epochs and 95.86% ± 4.89% for target-related epochs. Only artifact-free and correctly performed EEG epochs were included in the following analyses.

### Scalp ERP analysis

The CNV is a well-documented ERP marker for response preparation during the foreperiod^17–19, 27^. In the present study, cue-related epochs were averaged across the cue left and cue right trials with the 200 ms pre-cue interval as baseline to get the cue-related ERP for each electrode and participant. Since the CNV was typically observed over frontal-central scalp regions^17, 19, 20^, cue-related ERPs were averaged across frontal-central electrodes (Fz, Cz, FC1, FC2; see Fig. 2A). The amplitude of CNV was identified as the mean value within the late period of CTI (800–1200 ms post-cue).

In the present study, attentional modulation was measured by examining the amplitude of target-related N1 component, one of the most extensively reported electrophysiological measures of visual selective attention^20, 21, 26^. To this end, target-related epochs were grouped and averaged across trials according to the target location (left, right) and attention condition (attended, ignored) with the −200–0 ms pre-target interval as baseline, yielding the target-related ERP for each electrode and participant. Similar to our recent work^20^, target-related ERPs were averaged across the electrodes within two posterior ROIs where the N1 was typically observed (Fig. 2B). The amplitude of N1 was identified as the mean value within the time window of 170–210 ms post-target.

To analyze the N2 and P3 components, target-related epochs were grouped and averaged according to the type of targets (Go, NoGo) and attention condition (attended, ignored) with the 200 ms pre-target interval as baseline, yielding the target-related ERP for each electrode and participant. Since the P3 was typically observed over frontal-central scalp regions^22, 24, 43^, target-related ERPs were averaged across frontal-central electrodes (Fz, Cz, FC1, FC2). For the N2 component with a more anterior distribution (Fig. 4B), the amplitude was measured at the Fz electrode. The individual peak latencies of the N2 and P3 components were identified as the time points of the most negative and most positive voltages during pre-defined time windows, respectively: (i) Attend-Go and Attend-NoGo: 250–400 ms for the N2, 400–600 ms for the P3; (ii) Ignore-Go and Ignore-NoGo: 250–500 ms for the P3. We noted that the N2 was not observed for either Ignore-Go or Ignore-NoGo targets. The individual amplitudes of the N2 and P3 components were then identified as the mean values within a 40 ms time window centered around the individual peak latency. To minimize the interference of high-frequency noise, individual raw ERP was filtered into 0–15 Hz to get a reliable measurement of the individual peak latency. This filter was not applied for the measurement of amplitude, since the mean amplitude is less sensitive to high-frequency noise^22, 54^.

### Source localization analysis

The cortical three-dimensional distribution of current density can be reconstructed using standardized low-resolution brain electromagnetic tomography (sLORETA)^55^. Briefly, based on the individual topographical distribution of ERP amplitudes, sLORETA computes the standardized current density for each of 6239 voxels with 5 × 5 × 5 mm spatial resolution in the intracerebral volume in neuroanatomical Montreal Neurological Institute (MNI) space. sLORETA using 32 electrode configurations has been previously shown to be capable of localizing neural activity during Go/NoGo tasks by us^22^ and others^24, 56–58^.

In this study, individual sLORETA images for P3 were reconstructed within a 40 ms time window centered around the peak latencies: (i) 460 ms post-target for Attend-NoGo targets; (ii) 355 ms post-target for Ignore-Go and Ignore-NoGo targets. For the purpose of statistical comparison, individual sLORETA images were also reconstructed for ERPs elicited by Attend-Go targets during the time interval that was set up in correspondence to other types of targets. Voxel-by-voxel within-subject comparisons of the current density power of source images were performed using sLORETA to examine the possible differences between different experimental conditions.

### Statistical analysis

Repeated-Measures Analysis of Variance (ANOVA) and paired-samples *t*-test (2-tailed) were used to compare the ERP components between different types of targets using SPSS 16.0. Specifically, two-way ANOVA with Attention (attended, ignored) and Target (NoGo, Go) as within-subject factors was conducted for the P3 component, and a *t*-test was performed if an interaction was observed. The source data were tested in the sLORETA software. Specifically, empirical probability distributions were generated by 5000 random permutations and corresponding significance thresholds were assessed for the following statistical inferences. The SnPM (Statistical non-Parametric Mapping) approach was applied for the correction of multiple comparisons. Statistical significance was accepted for values of *p* < 0.05. All results were presented as mean ± SEM (standard error of the mean) unless otherwise specified.

### Data availability statement

The datasets analyzed during the current study are available from the corresponding author on reasonable request.
